# MCM10 is a Prognostic Biomarker and Correlated With Immune Checkpoints in Ovarian Cancer

**DOI:** 10.3389/fgene.2022.864578

**Published:** 2022-05-19

**Authors:** Zhenzhen Wu, Yueyuan Wang, Juan Li, Huiling Wang, Xunyuan Tuo, Jing Zheng

**Affiliations:** ^1^ Department of Gynecology, Gansu Provincial Maternity and Child-Care Hospital, Lanzhou, China; ^2^ Department of Pathology, Gansu Provincial Maternity and Child-Care Hospital, Lanzhou, China; ^3^ Department of Gynecological Oncology, Gansu Provincial Maternity and Child-Care Hospital, Lanzhou, China

**Keywords:** ovarian cancer, Mcm10, bioinformatics, prognosis biomarkers, immune checkpoints

## Abstract

**Background:** Microchromosome maintenance protein 10 (MCM10) is required for DNA replication in all eukaryotes, and it plays a key role in the development of many types of malignancies. However, we currently still do not know the relationship between MCM10 and ovarian cancer (OV) prognosis and immune checkpoints.

**Methods**: The Gene Expression Profiling Interactive Analysis and Tumor Immunology Estimation Resource (TIMER) databases were used to investigate MCM10 expression in Fan cancer. The Kaplan-Meier Plotter and PrognoScan were used to assess the relationship between MCM10 and OV prognosis. The LinkedOmics database was used to analyze the MCM10 co-expression network and explore GO term annotation and the KEGG pathway. The relationship between MCM10 expression and immune infiltration in OV was investigated using the Tumor Immunology Estimation Resource database. cBioPortal database was used to explore the relationship between MCM10 expression and 25 immune checkpoints. Finally, quantitative real-time polymerase chain reaction (qRT-PCR) was performed to detect MCM10 expression. The prognosis was also analyzed by distinguishing between high and low expression groups based on median expression values.

**Results**: The results of the three data sets (220,651_s_at, 222,962_s_at and 223,570_at) in KM Plotter all indicated that the overall survivalof the high MCM10 expression group was lower than that of the low expression group OV, and the results of GSE9891 also reached the same conclusion. The expression level of MCM10 was negatively correlated with B cells and CD8+T cells, and positively correlated with CD4+T Cells and Macrophages. GO term annotation and KEGG pathway analysis showed that the co-expressed genes of MCM10 were mainly enriched in cell cycle and DNA replication. The alterations in MCM10 coexisted statistically with the immune checkpoints CTLA4, TNFSF4, TNFSF18, CD80, ICOSLG, LILRB1 and CD200. PCR results displayed that MCM10 was highly expressed in OV tissues, and the increased expression of MCM10 was significantly associated with poor overall survival.

**Conclusion**: These results demonstrated that high expression of MCM10 was associated with poor prognosis in OV and correlated with immune checkpoints.

## Introduction

Ovarian cancer (OV) is a common but fatal gynaecological malignancy. Although the mortality rate from OV has declined over the past 40 years as medical care has improved, it still remains the second leading cause of death from gynaecological cancers in women and the eighth leading cause of death in women (Irusta, 2021). The current treatment for OV is mainly surgical resection and platinum-based chemotherapy, the combination of which usually brings good outcomes, with the addition of anti-angiogenic agents usually for poorly operated and stage IV patients (Lheureux et al., 2019). Despite many efforts to treat OV, the prognosis still remains poor due to the recurrence and metastasis of OV. Thus, identifying novel regulators as diagnostic and therapeutic targets for OV is still urgently required.

Minichromosome maintenance (MCM) proteins are essential for the initiation of DNA replication, and it has been detected to be overexpressed in various cancer tissues, including lung squamous cell carcinoma (Wu et al., 2018), breast cancer (Juríková et al., 2016), glioma (Cai et al., 2018), hepatocellular carcinoma (Liu et al., 2018), etc. As an important player in the initiation pathway of DNA replication, Minichromosome maintenance 10 (MCM10) was first identified in a yeast genetic screen and only presents in eukaryotes (Aves et al., 1998). At the same time, the involvement of MCM10 has also been found in DNA elongation, bolstering the activity of the CMG helicase on bypassing replication blocks (Langston et al., 2017; Lõoke et al., 2017) and promotion of replication fork progression and stability (Baxley and Bielinsky, 2017). In addition to the above group roles, MCM10, like other MCM family proteins, is abnormally expressed in various tumors and associated with prognosis. The overexpression of MCM10 is thought to promote the abnormal proliferation of prostate cancer (PC) cells and associated with poor prognosis of PC (Cui et al., 2018). Meanwhile, it is positively related to poor prognosis in breast cancer (Yang and Wang, 2019). In glioma, the knockdown of MCM10 in glioma cells resulted in decreased cell proliferation, migration and invasion (Kang et al., 2020). However, we have not found many articles on the relationship between MCM10 and OV. Based on the close relationship between MCM10 and various malignant tumors, we have reasons to believe that MCM10 is a potential prognostic marker.

Increasing research results proved that immunotherapy is a very promising therapeutic method in the treatment of malignant tumors, among which the blockade of immune checkpoints has displayed significant efficacy in various types of tumors (Topalian et al., 2016). Interfering with Cytotoxic T lymphocyte (CTLA-4) and ProgrammedDeath-1 (PD-1) reportedly has clinical benefits in several human cancers (Odunsi, 2017), so the characterizing associations between MCM10 and immune checkpoints will potentially enhance OV treatment.

In this study, the online tools TIMER and GEPIA were used to explore the expression of MCM10 in various malignancies. The prognostic value of MCM10 expression in OV was determined using the Kaplan-Meier Plotter and PrognoScan databases. LinkedOmics database was used to view genes and pathways associated with MCM10. The cBioPortal database was used to visualize and compare genetic alterations and explore the association between MCM10 and 25 immune checkpoints. Finally, qRT-PCR was used to detect the expression of MCM10 and analyze the relationship between its expression and prognosis. Our findings revealed an significant role for MCM10 in OV expression and prognosis, and also elucidated the relationship between MCM10 and multiple immune checkpoints.

## Materials and Methods

### Tissue Samples

A total of 22 cancerous and 50 paracancerous ovarian tissue samples were obtained during surgery. The study was approved by the jurisdictional clinical research ethics committees. All patients consented to the study.

### qRT-PCR

Frozen tissues (100 mg) were ground into powder in liquid nitrogen, and then suspended in 1 ml TRIZOL Reagent (Invitrogen, United States of America), Total RNA was extracted using TRIZOL reagent. RNA was quantified using a spectrophotometer (Beckman, United States of America). RNA template and random primers were incubated at 70°C for 10 min to melt the secondary structure within the template, and cooled on ice for more than 2 min. Then the complete reaction mixture was incubated at 30°C for 10 min, 42°C for 60 min and 70°C for 15 min. PCR was performed in a total volume of 25 μl containing 1 μl of reverse-transcribed cDNA. After an initial incubation at 94°C for 5 min, the reaction mixtures were subjected to 35 cycles of amplification using the following protocols: 94°C for 45 s, 55°C for 45 s and 72°C for 45 s, followed by a final extension step at 72°C for 7 min. PCR products were analyzed by 1.2% agarose gel electrophoresis and stained with GoldView nucleic acid dye. Real-time RT-PCR was performed using ABI PRISM 7500 Sequence Detection System instrument and software (Applied Biosystems, United States). The relative expression level of MCM10 was measured using SYBR Green I dye-based method. The results were normalized to the expression of glyceraldehyde-3-phosphate dehydrogenase (GAPDH). The Ct values of the amplified products were used in conjunction with the 2^−ΔΔCt^ method to analyze the data (Schmittgen and Livak, 2008; Zhang et al., 2010). The primers used were GAPDH: 5′-AGA​AGG​CTG​GGG​CTC​ATT​TG-3′ (F), 5′-AGG​GGC​CAT​CCA​CAG​TCT​TC-3′ (R); MCM10:5′-CACAGAAATGAACAAGAA-3′(F),5′-AATAAGAACAAGGACACA-3′(R); Primers were synthesized by BGI Company.

### GEPIA Database Analysis

GEPIA (http://gepia.cancer-pku.cn/index.html) is a recently developed bioinformatics platform that incorporates genotype tissue expression data from 9,736 tumors and 8,587 normal samples. In the current study, the “Expression DIY” component was used to analyze the EFNA1 expression levels in a variety of cancers and adjacent tissues, and *p* < 0.05 was used as the screening threshold significance level (Tang et al., 2017).

### TIMER Database

TIMER (https://cistrome.shinyapps.io/timer/) is a tumor immunity database, including 10,897 cancer samples from the TCGA database, together with an abundance of tumor-infiltrating immune cells (TIICs) based on a deconvolution method from gene expression profiles. In the present study, the “Gene” module was applied to analyze the correlations between EFNA1 expression and immune cell infiltration. The immune cells analyzed included CD4^+^ T cells, CD8^+^ T cells, B cells, neutrophils, macrophages, and dendritic cells (DCs). The “Correlation” module of TIMER was used to analyze the associations between EFNA1 and other prognosis-related immune cell markers, including CD8^+^ T cells, all T cells collectively, B cells, monocytes, tumor-associated macrophages (TAMs), M1 and M2 macrophages, neutrophils, natural killer (NK) cells, and DCs (Li et al., 2017).

### Survival Analysis and Prognostic Evaluation

Kaplan–Meier Plotter (http://kmplot.com/analysis/) and PrognoScan (http://dna00.bio.kyutech.ac.jp/PrognoScan/index.html) were used for prognostic analyses. Kaplan–Meier Plotter evaluated the prognostic significance of MCM10 mRNA expression in OV. Patients’ samples were divided into two groups based on the median MCM10 expression level, and the overall survival (OS) of patients with OV was analyzed. The examination probe ID was used for MCM10 was 220,651_s_at, 222,962_s_at and 223,570_at. The log-rank *p*-value and hazard ratio (HR) with 95% confidence intervals were calculated. The PrognoScan database mainly collects clinical prognostic information derived from 14 cancers from GEO (Gene Expression Omnibus) and various laboratories, and then applies a minimum *p* value approach in analyses. In the current study, it was used to analyze the prognostic value of MCM10 in OV and adjust the threshold to a Cox *p* value (Mizuno et al., 2009; Lánczky and Győrffy, 2021).

### cBioPortal Database

cBioPortal for Cancer Genomics (http://cbioportal.org) is a large repository of genomics datasets. In the present study, cBioPortal was used to visualize and compare the changes in EFNA1 and immune checkpoints in OV. The correlations between MCM10 and immune checkpoints were also investigated. The immune checkpoints analyzed included PD-L1 (CD274), PD-L2 (PDCD1LG2), CD80, CD86, VTCN1, VSIR, HHLA2, TNFRSF14, PVR, CTL4, CD112 (NECTIN2), CD200, LGALS9, ICOSLG, TNFSF9, TNFSF4, CD70, TNFSF18, CD48, CTLA4, CD276, LILRB1, LILRB2, HAVCR2, CD47 and TNFRSF9(CD137) (Wu et al., 2019).

### COSMIC Database Analysis of MCM10 Mutations in OV

The Catalogue of Somatic Mutations in Cancer (COSMIC) (https://cancer.sanger.ac.uk) is the most detailed and comprehensive resource to explore the effects of somatic mutations in human cancer. COSMIC database Contains 6 million coding mutations (Tate et al., 2019). Ovary in the“tissue distribution”and “mutation distribution”were chosen.

### LinkedOmics Database Analysis of MCM10-Related Pathways

The LinkedOmics (http://www.linkedomics.org) database includes 32 cancer types from TCGA project and 11,158 patients with multiple omics and clinical data. It is also the first multi-omics database that integrates mass spectrometry–based global proteomics data generated by the Clinical Proteomics Cancer Analysis Alliance on selected TCGA tumor samples (Vasaikar et al., 2018). The differentially expressed genes related to MCM10 were screened from the TCGA OV cohort through the LinkFinder module in the database, and the correlations of the results were presented in volcano plots and heat maps, respectively, by Pearson correlation coefficient test. Gene Set Enrichment Analysis (GSEA) in the LinkInterpreter module performed functional module analysis of the Gene Ontology Biological Process (GO_BP), Kyoto Encyclopedia of Genes and Genomes (KEGG) pathways. And 0.05 was considered as the *p*-value cutoff and the Spearman correlation test was conducted to analyze the results statistically.

### Statistical Analysis

All data were expressed as means and standard deviations, and SPSS 19.0 software (SPSS Inc. Chicago, IL, United States) was the statistical analysis tool used. The student’s *t*-test and one-way analysis of variance were carried out to analyze the differences between groups. *p* < 0.05 was deemed to indicate the statistical significance. All experiments were conducted in triplicate as a minimum. The Kaplan–Meier Plotter and GEPIA results are presented as hazard ratios (HRs) and *p* values, and PrognoScan results are presented as Cox *p* values.

## Results

### mRNA Expression Levels of MCM10 in Different Types of Human Cancers

**FIGURE 1:**
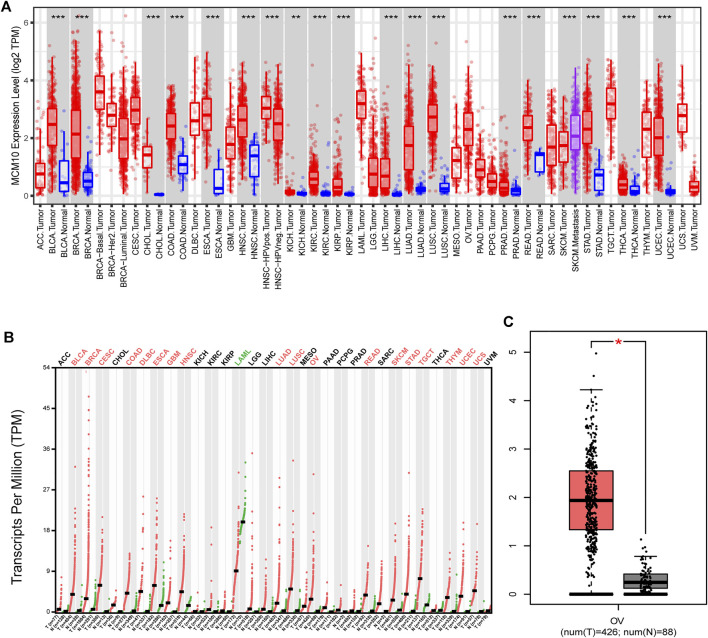
MCM10 expression levels in different types of human cancers. **(A)** MCM10 expression in different tumor types in TIMER. **(B)** MCM10 expression in different tumor types in GEPIA. **(C)** Box plots comparing MCM10 expression in OV and unpaired normal tissues in GEPIA based on analysis of variance method (TCGA tumor versus TCGA normal + GTEx normal).*p < 0.05, **p < 0.01, ***p < 0.001.

To evaluate the differences of MCM10 expression in tumor and normal tissues, the MCM10 mRNA levels in tumor and normal tissues of patients with multiple types of cancer were analyzed using the GEPIA and TIMER database. MCM10 expression was higher in BLCA, BRCA, CHOL, COAD, HNSC, KICH, KIRC, KIRP, LIHC, LUAD, PRAD, READ, STAD, THCA, and UCEC, compared with normal tissues in TIMER (Figure 1A). In GEPIA, high expression in BLCA, BRCA, CESC, COAD, DLBC, ESCA, GBM, HNSC, LUAD, LUSC, OV, READ, SKCM, STAD, TGCT, THYM, UCEC and UCS was observed. In addition, lower expression was observed in LAML (Figure 1B). MCM10 was highly expressed in OV (Figure 1C).

### Relationships Between MCM10 and the Prognosis of OV

**FIGURE 2:**
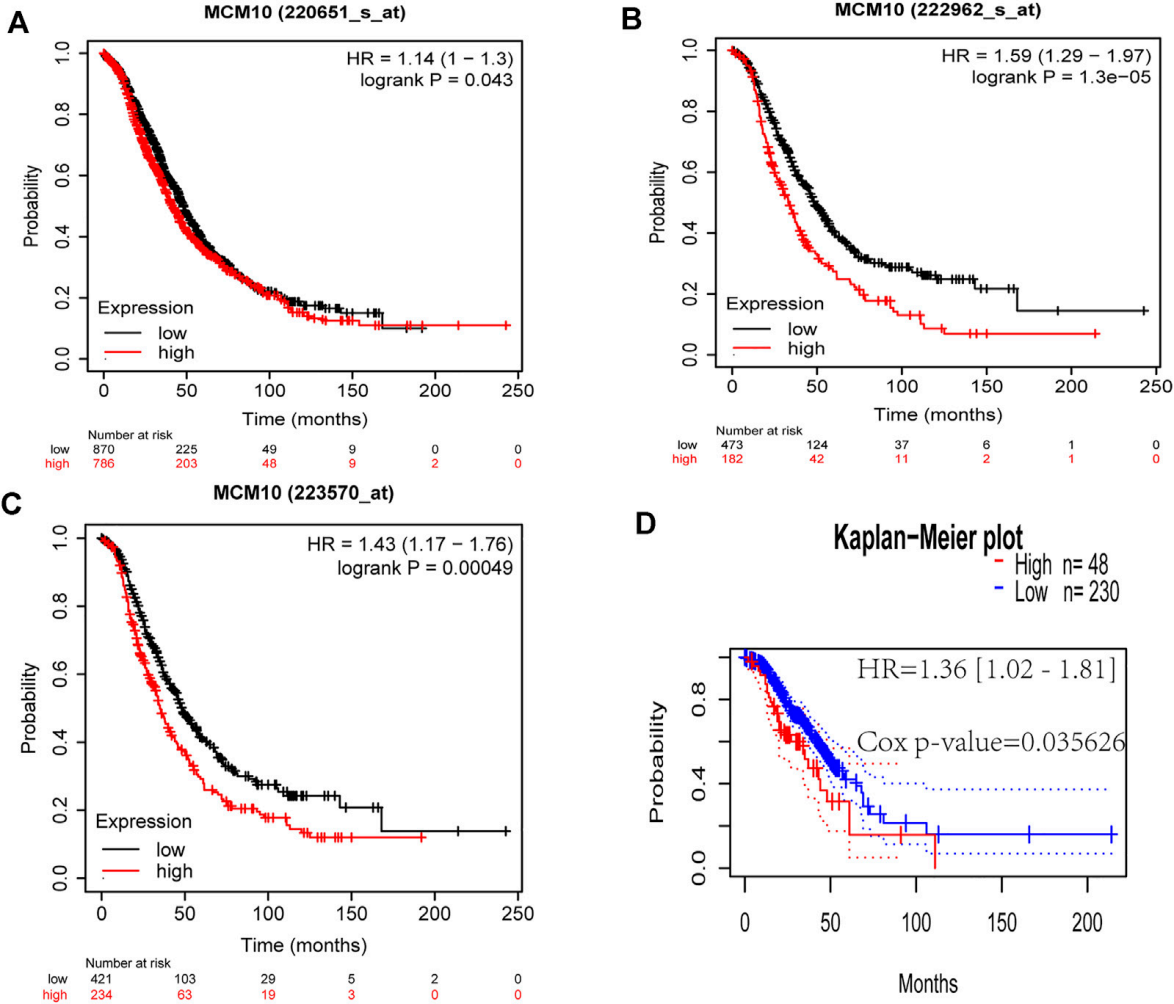
Correlations between MCM10 expression and prognostic indicators in OV. **(A–C)** Correlations between MCM10 and OV prognoses in the Kaplan–Meier Plotter database from different expression callers (220,651_s_at,222,962_s_at, 223,570_at). **(D)** Survival curve from PrognoScan analysis for OS of patients with OV. HR = hazard ratio.

The prognostic value of MCM10 expression in OV was evaluated using Kaplan-Meier plots and PrognoScan. The expression of MCM10 was significantly associated with the prognosis of OV patients. In analyses with the Kaplan-Meier plots, the OS 220651_s_at [HR = 1.14 (1 -1.3), *p* = 0.043], 222,962_s_at [HR = 1.59 (1.29 -1.97), *p* = 1.3e-05] and 223,570_at [HR = 1.43 91.17 -1.760, *p* = 0.00049] of OV patients with high MCM10 expression (Figures 2A–C) values were significantly lower than those of patients with low MCM10 expression. In PrognoScan database analysis of the prognostic potential of MCM10 in OV, high MCM10 expression in the GSE9891 cohort was associated with poor OS [HR = 1.36 (1.02 -1.810, *p* = 0.035626] (Figure 2D).

### MCM10 Co‐Expression Network in OV

**FIGURE 3:**
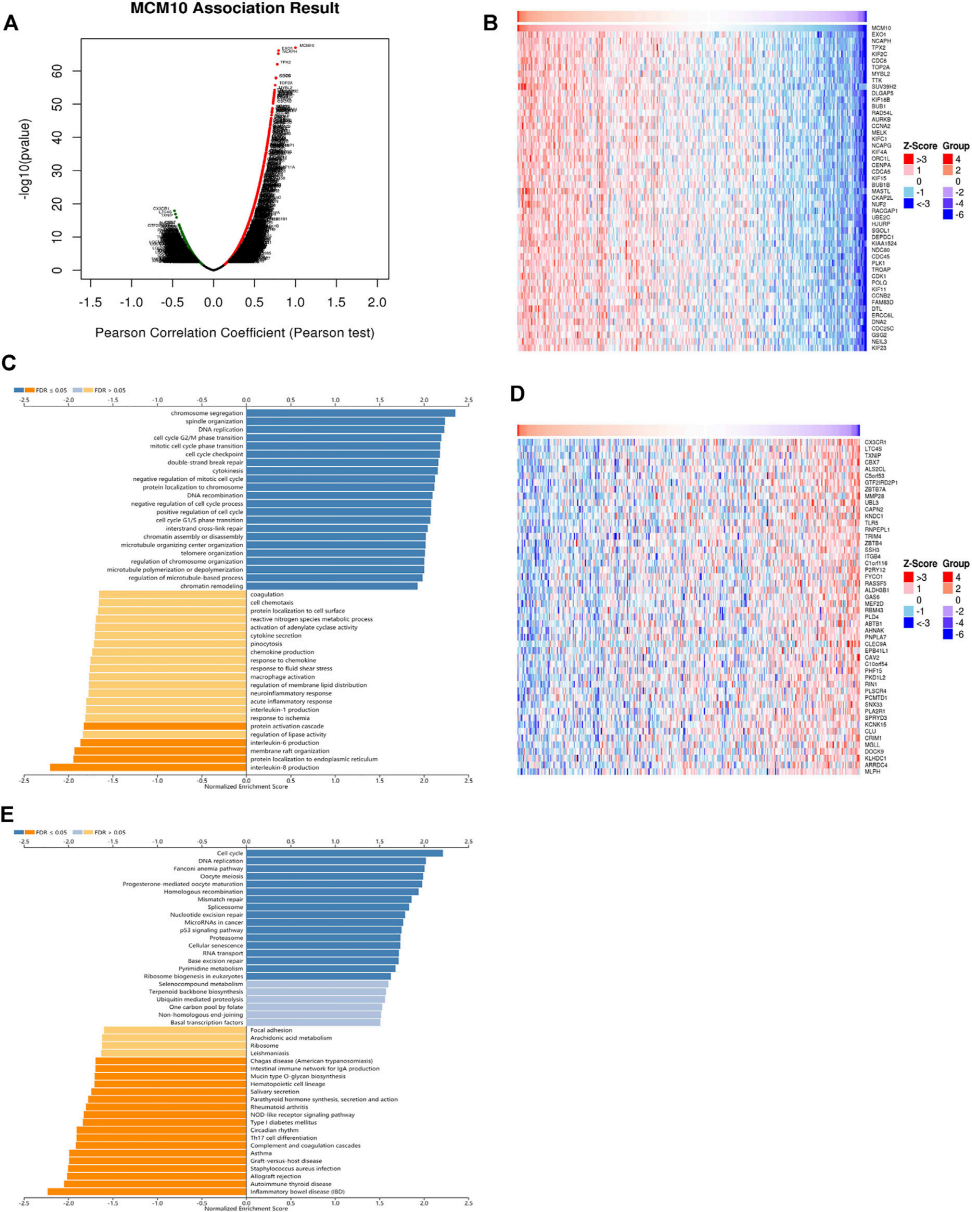
The co‐expression genes with MCM10 from the LinkedOmics database in OV. **(A)** The whole significantly associated genes with MCM10 distinguished in OV cohort. **(B–D)** Top 50 genes positively and negatively related to MCM10 in OV **(C,E)** GO annotations and KEGG pathways of CLEC10A in LUAD cohort.

To understand the biological function of MCM10 in OV, the LinkFinder module in the LinkedOmics portal was used to examine the co-expression pattern of MCM10 in TCGA-OV. As shown in Figure 3A genes positively correlated with MCM10 were dark red dots, and 3,613 genes negatively correlated with MCM10 were dark green dots. Figures 3B–D represents the top 50 genes associated and negatively associated with the MCM10 signature, respectively. GO term annotation proved that the co‐expressed genes of MCM10 join mainly in chromosome segregation, spindle organization, DNA replication, cell cycle G2/M phase transition, mitotic cell cycle phase transition, cell cycle checkpoint, double-strand break repair, cytokinesis, negative regulation of mitotic cell cycle and protein localization to chromosome, etc (Figure 3C). KEGG pathway analysis indicated the enrichment in Cell cycle, DNA replication, Fanconi anemia pathway, Oocyte meiosis, Progesterone-mediated oocyte maturatio, Homologous recombination, Mismatch repair, Asthma, Graft-versus-host disease, and *Staphylococcus aureus* infection, etc (Figure 3C). It was found that GO terms and KEGG pathways were more concentrated in cell cycle and DNA replication.

### Mutation of MCM10 in OV

**FIGURE 4:**
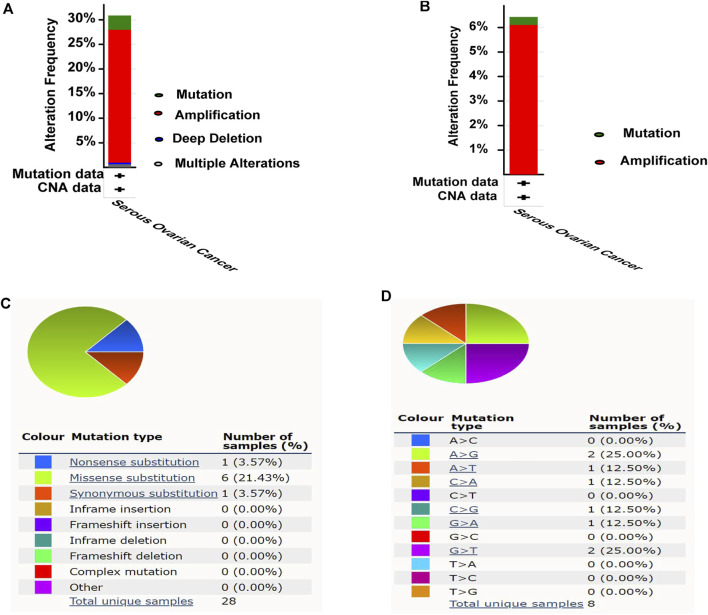
Mutation analysis of MCM10 in OV. **(A)** Mutation frequency of MCM10. **(B)** Mutation frequency of MCM10. **(C–D)** The mutation types of MCM10 in OV by Catalogue of Somatic Mutations in Cancer (COSMIC) database.

The cBioPortal was used to explore the mutation status of MCM families (MCM2, MCM3, MCM4, MCM5, MCM6, MCM7, MCM8, MCM9, MCM10 and MCMBP). Totally, 30.87% (96/311) of patients had genetic alterations (Figure 4A), of which MCM10 accounted for 6.43% (20/311), and the amplification was the most frequent mutation (Figure 4B). In COSMIC, we further assessed the mutation type of MCM10, Missense substitutions occurred in approximately 21.43% of the samples, synonymous substitutions occurred in 3.57% of the samples, and nonsense substitutions occurred in 3.57% of the samples (Figure 4C). The substitution mutations mainly occurred at A > G (25.00%) and G > T (25.00%), followed by A > T (12.50%), C > A (12.50%), C > G (12.50%), and G > A (12.50%) (Figure 4D).

### Correlation of MCM10 With Immune Infiltration and Immune Checkpoints

**FIGURE 5:**
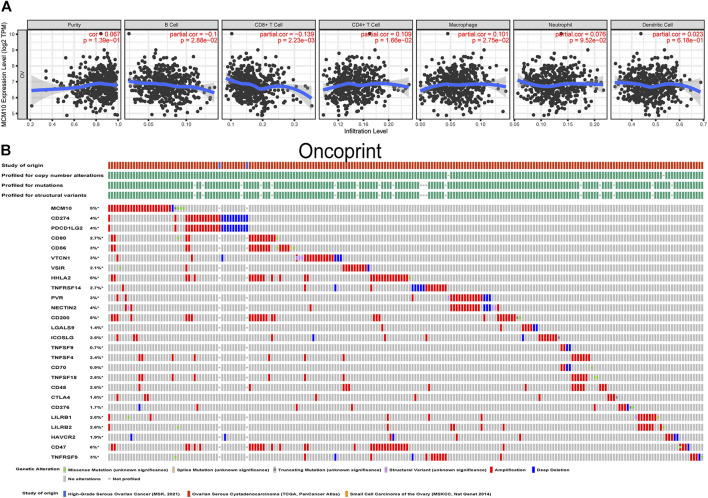
Correlation of MCM10 with immune infiltration and immune checkpoints. **(A)** the correlations between MCM10 and immune cell infiltrations from TIMER. **(B)** Landscape of MCM10 and immune checkpoint alteration in OV from cBioPortal.

The presence of immune infiltration within tumors can generate important biomarkers to predict the prognosis of tumor patients, with impacts on radiotherapy, chemotherapy and therapy. Therefore, it is cardinal to study the relationship between MCM10 and immunity. We used the “Gene” module in TIMER for database search, entered the target gene MCM10, and selected OV. This module displays infiltration results, including TIMER, EPIC, MCP-COUNTER, CIBERSORT, CIBERSORT-ABS, QUANTISEQ, XCELL, QUANTISE and TIDE. The expression of MCM10 was positively correlated with CD4+T Cells (cor = 0.109, *p* = 0.0166) and Macrophages (cor = 0.101,*p* = 0.0275), and was positively correlated with B cells(cor = -0.1,*p* = 0.0288) and CD8+T Cells(cor = -0.139, *p* = 0.00223) (Figure 5A). The relationship between genetic changes in the MCM10 gene and 25 immune checkpoints was explored. Three datasets (MSK, TCGA and MSKCC), including 612 samples, were selected, and genomic studies revealed that MCM10 was involved in the alteration of OV immune checkpoints. The alterations of MCM10 and immune checkpoints in OV were visualized in a compact manner. In OV, the Genetic Alteration of MCM10 was 5% and mainly concentrated in Amplification. There was a part of Missense Mutation, second only to 6% of the immune checkpoint CD47, which was equal to HHLA2 and CD200. This indicated that MCM10 had a high mutation rate during the progression of OV (Figure 5B). Then, the association between MCM10 and each immune checkpoint was examined. Notably, the alterations in MCM10 showed statistically significant coexistence rather than rejection with the immune checkpoints CTLA4, TNFSF4, TNFSF18, CD80, ICOSLG, LILRB1 and CD200 (Table 1). These findings strongly suggested that MCM10 is a potential co-regulator of the OV immune checkpoint.

**TABLE 1:** Mutual-exclusivity analysis between MCM10 and multiple-immune checkpoints in ovarian cancer.

A	B	Neither	A not B	B not A	Both	Log2 Odds ratio	*p*-value	q-value	Tendency	Significant
MCM10	CTLA4	477	25	6	3	>3	0.01	0.154	Co-occurrence	MCM10
MCM10	TNFSF4	474	25	9	3	2.66	0.023	0.329	Co-occurrence	MCM10
MCM10	TNFSF18	473	25	10	3	2.505	0.029	0.379	Co-occurrence	MCM10
MCM10	CD80	472	25	11	3	2.364	0.036	0.401	Co-occurrence	MCM10
MCM10	ICOSLG	472	25	11	3	2.364	0.036	0.401	Co-occurrence	MCM10
MCM10	LILRB1	472	25	11	3	2.364	0.036	0.401	Co-occurrence	MCM10
MCM10	CD200	461	24	22	4	1.804	0.047	0.506	Co-occurrence	MCM10
MCM10	NECTIN2	465	25	18	3	1.632	0.101	0.819	Co-occurrence	MCM10
MCM10	LILRB2	472	26	11	2	1.723	0.156	0.928	Co-occurrence	MCM10
MCM10	CD86	467	26	16	2	1.167	0.258	0.928	Co-occurrence	MCM10
MCM10	PVR	467	26	16	2	1.167	0.258	0.928	Co-occurrence	MCM10
MCM10	CD274	465	26	18	2	0.991	0.301	0.928	Co-occurrence	MCM10
MCM10	PDCD1LG2	465	26	18	2	0.991	0.301	0.928	Co-occurrence	MCM10
MCM10	HAVCR2	475	27	8	1	1.137	0.4	0.928	Co-occurrence	MCM10
MCM10	HHLA2	460	26	23	2	0.621	0.404	0.928	Co-occurrence	MCM10
MCM10	VSIR	473	27	10	1	0.809	0.465	0.928	Co-occurrence	MCM10
MCM10	CD276	473	27	10	1	0.809	0.465	0.928	Co-occurrence	MCM10
MCM10	TNFRSF14	472	27	11	1	0.668	0.495	0.928	Co-occurrence	MCM10
MCM10	CD47	454	26	29	2	0.268	0.519	0.928	Co-occurrence	MCM10
MCM10	CD48	471	27	12	1	0.54	0.524	0.928	Co-occurrence	MCM10
MCM10	TNFRSF9	469	27	14	1	0.311	0.576	0.928	Co-occurrence	MCM10
MCM10	LGALS9	475	28	8	0	<-3	0.635	0.928	Mutual exclusivity	MCM10
MCM10	VTCN1	465	27	18	1	-0.064	0.72	0.928	Mutual exclusivity	MCM10
MCM10	CD70	478	28	5	0	<-3	0.754	0.928	Mutual exclusivity	MCM10
MCM10	TNFSF9	479	28	4	0	<-3	0.798	0.928	Mutual exclusivity	MCM10

### Validation of MCM10 in OV Tissues

**FIGURE 6:**
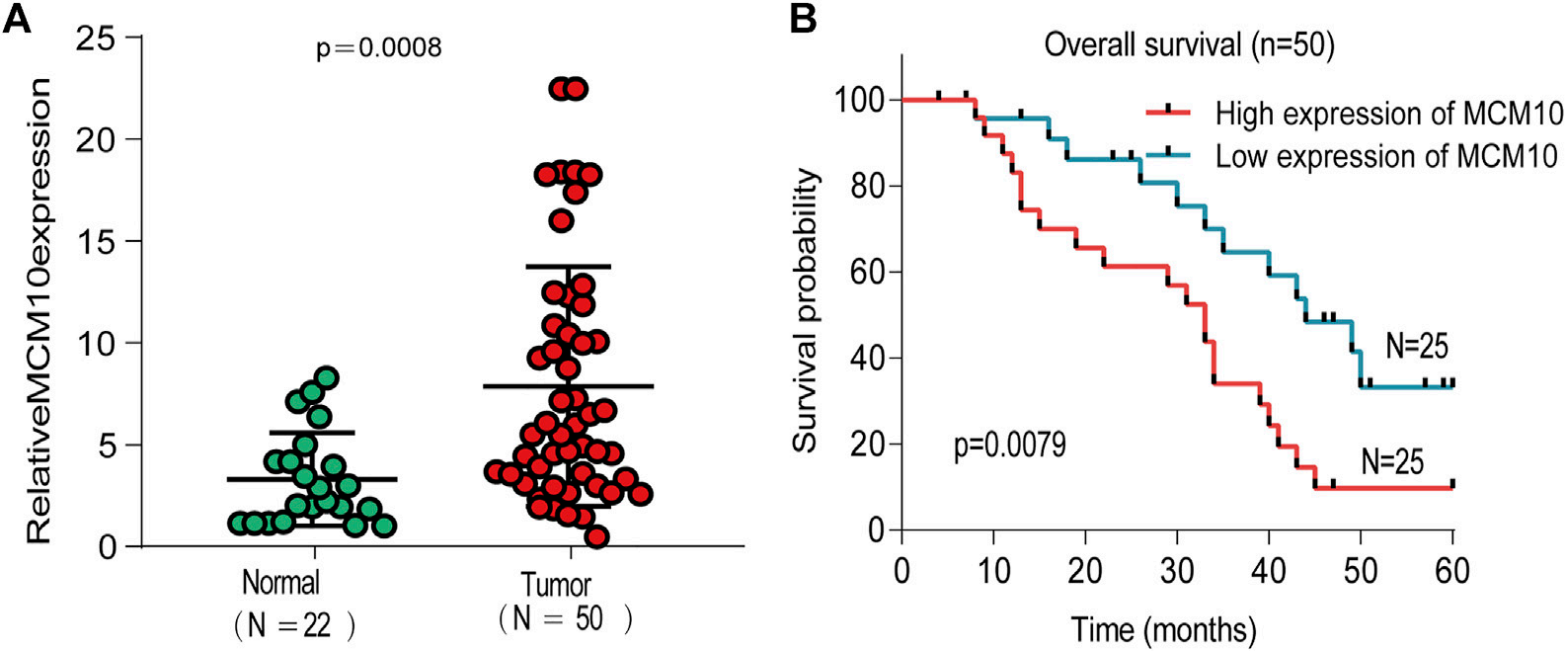
Expression of MCM10 in Independent OV Cohorts. **(A)** qRT-PCR showed that MCM10 expression was up-regulated in OV tissues. **(B)** High MCM10 expression have worse prognosis in OV patients.

To explore the expression of MCM10 in OV, qRT-PCR validation was chosen. The results suggested that the expression of MCM10 was elevated in OV (Figure 6A). According to the median value of expression, the patients were divided into high and low expression groups to judge the prognosis of patients (Figure 6B). The results illustrated that OV patients with high MCM10 had a worse prognosis.

## Disscussion

One of the characteristics of tumor cells is unlimited proliferation. Therefore, many proteins related to DNA replication have been considered as potential cancer biomarkers, including MCM protein (Yu et al., 2020). MCM protein has been considered as a biomarker of dysplasia and tumor (Wang et al., 2020). There are several hypotheses about the mechanism of MCM dysregulation leading to tumorigenesis. The first is genomic instability (GIN), because the formation of cancer cells is caused by the accumulation of mutations in oncogenes and tumor suppressor genes. In many studies, GIN caused by MCM mutation has been proved to be related to the occurrence of malignant tumors. (Chuang et al., 2010). Secondly, damage to MCM induces replicative stress, a critical step in the initiation of the oncogenic process (Gaillard et al., 2015). Finally, the study found that MCM family proteins participate in the progression of cell cycle pathways. For example, knockdown of MCM2 reduces the expression of cyclinD1, cyclinA and CDK4, knockdown of MCM3 reduces the expression of cyclinA, knockdown of MCM6 causes CyclinA, CyclinB1,CyclinD1, silencing of MCM7 reduces cyclinD1, cyclinE2 and CDK2, and down-regulation of cyclinD1 in breast cancer cells with MCM10 knockdown (Zhang et al., 2015; Qiu et al., 2017; Liu et al., 2018; Yang and Wang, 2019). These results all suggest that mutations in MCM proteins cause changes in various cyclins, and ultimately aberrant cell cycle progression leads to tumorigenesis. There are few studies on MCM10 in the MCM protein family, especially its relationship with OV, so this study focused on this gene, which has not received much attention but is extremely significant. MCM10 acts as an vital scaffold for DNA replication and protection against replication stress under normal conditions. However, under pathological conditions, MCM10 is frequently deregulated, and gene amplification and overexpression are very common in cancer (Baxley and Bielinsky, 2017). Our results also demonstrated that the changes of the MCM10 gene in OV are mainly concentrated in the amplification, and it was discovered that most of the mutations in MCM10 are missense mutations (93%), and the rest are roughly divided into splicing mutations (3.7%) and nonsense mutations (3.2%) (Gao et al., 2013; Kang et al., 2013), which is consistent with our findings. The mechanism by which MCM10 causes OV, in addition to the aforementioned, may also be related to the specific relationship between MCM10 and female ovaries, where MCM10 is highly expressed in adult female ovaries (Graveley et al., 2011). Based on this, Reubens et al. conducted further research and believed that MCM10 plays a unique biological role in the development or maintenance of the female germline (Reubens et al., 2015). So the mutation of MCM10 may be another cause of OV progression. These data clearly indicated that MCM10 changes in the cancer genome, but whether these changes are the causes or the results of OV still needs further studies to confirm.

Our results proved that the expression of MCM10 in various malignant tumors is different from that in normal tissues, except for the low expression of LAML in the GEPIA database, because the data in the TIMER database are all from TCGA, and normal control samples of some malignant tumors are insufficient, but MCM10 is highly expressed in malignant tumors with differential expression. Since we only obtained high expression of MCM10 in OV from GEPIA, we further verified the expression of MCM10 in OV by qRT-PCR, and it was found that the expression of MCM10 in OV was higher than that in normal ovarian tissue. Subsequently, the relationship between the expression of MCM10 and the prognosis of OV patients was examined in four databases, and it was obvious that the prognosis of OV patients with high expression of MCM10 was worse. After being divided into high and low expression, the patients in the high expression group also had a poor prognosis. These findings all demonstrated that MCM10, as a promising prognostic biomarker, is increased in OV.

Having verified the differential expression and prognostic potential of MCM10 in OV, in order to better serve the clinic, we next explored the possible pathogenic mechanism of MCM10. In order to test our previous speculation about the pathogenic mechanism of MCM10, the effects of MCM10 changes in OV on the transcriptome were explored, and it was found that 4,297 positively correlated genes and 3,613 negatively correlated genes were changed accordingly, which suggested that the alterations in MCM10 have broad impacts on the transcriptome. The analysis results of GO and KEGG both illustrated that the pathway enrichment of other gene changes caused by MCM10 changes mainly concentrated in the related pathways of cell cycle and DNA replication. Since MCM10 itself participates in DNA replication, it is not surprising that the changes are mainly concentrated in DNA replication. However, the changes of MCM10 are closely related to the cell cycle, which may be closely related to the cell cycle, because the DNA replication process depends on the regulation of the cell cycle (Tachibana et al., 2005). Previous studies suggested that MCM10 may be part of a high-priority group of genes that may promote cell cycle-related processes in cancer cells (Cerami et al., 2012). A series of cell cycle checkpoint inhibitors targeting OV are already under development or clinical trials (Pujade-Lauraine, 2017). For example, cells initiate multiple responses to protect the genome and ensure survival against DNA damage, and unsuccessful DNA damage repair can lead to mitotic abnormalities and cell death (Lin et al., 2017). High-grade serous ovarian cancer (HGSOC) relies heavily on G2 checkpoint blockade to promote DNA damage repair, which is a process that opens up a new perspective for the treatment of OV (Haynes et al., 2018). If blocking MCM10 can affect both DNA replication and cell cycle, why not? Therefore, the combined treatment of DNA replication and cell cycle intervention may benefit OV patients.

People who care about OV treatment know that OV treatment cannot be cured by single-agent therapy because the results of single-agent studies on OV so far have been disappointing (Disis et al., 2019). The results of the drug combination may have some benefit, but the results are not better than the historical control (Lee et al., 2019). Therefore, it is necessary to explore a new combination therapy method. Tumor immunotherapy is considered as a new and potential tumor treatment method. The infiltration state of tumor-associated immune cells *in vivo* together constitutes the immune microenvironment of tumor cells, and these immune cells may have tumor antagonism or tumor promotion (Jain, 2021). Our results demonstrated that the expression level of MCM10 is negatively correlated with B cells and CD8+T Cells, and positively correlated with CD4+T Cells and Macrophages. This suggests that MCM10 plays a particular role in the immune infiltration of OV. In addition to the critical role of the immune microenvironment in anticancer immunity, another most popular approach in immunotherapy is immune checkpoint blockade (Huang et al., 2020). PD-1/PD-L1 and CTLA-4 are considered as the most principal immune checkpoints at present. In the past decade, great progress has been made in the field of immune checkpoint-related researches. immune checkpoint blockade (ICB) has been very successful in this type of cancer (Havel et al., 2019). However, since the current FAD-approved immune checkpoint inhibitors (ICI) are all monoclonal antibodies (mAbs), there are many shortcomings. Therefore, the therapeutic effects of ICB on OV are still limited. The study on small molecule inhibitors to eliminate the limitations of mAbs is a new direction for ICB therapy (Zhang et al., 2020; Zhang et al., 2021). More and more evidence showed that small molecule inhibitors that target oncogenic play a role far beyond the biological behavior of tumors. Some studies have found that some small molecule inhibitors directly participate in mediating the tumor microenvironment and promoting tumor cell death (Chen et al., 2019; Ziogas et al., 2021). For example, the inhibitors of CDK4/6 can synergize with PD-1 blockade and benefit the treatment of OV (Zhang et al., 2020). It has been revealed that the matrix metalloproteinases (MMPs) inhibitor, SB-3CT, can enhance the effects of PD-1 and CTLA-4 blockade in primary and metastatic tumors in studies (Ye et al., 2020). Small-molecule inhibitor JQ1 targeting BET bromodomains reduces PD-L1 expression, while attenuating progression in PC models (Mao et al., 2019). All of the above studies have proved that ICI combined with small molecule inhibitors is an effective way to address the shortcomings of current ICIs such as low oral availability, long tissue retention time and poor membrane permeability. Changes in seven ICIs occurred simultaneously, and considering that MCM10 is a member of a high-priority gene, the combination of its small-molecule inhibitor and ICI greatly benefits OV patients.

In our study, the expression and prognosis of MCM10 in OV were analyzed by bioinformatics methods, and the related pathways of MCM10 that were related to immune infiltration and immune checkpoints were analyzed. However, inevitably, our tests have certain limitations. Firstly, the number of patients enrolled in our validation experiment is relatively small, and we will update the number of patients in the later stage. Secondly, we only propose possible pathogenic pathways, and further experimental verification is needed, for example, we could test the effects of an artificial knockdown of MCM10 expression on tumor size and progression in cell models, validate qPCR results from tumor samples with Western blots, test for differences between MCM10 expression in CD8 mutant backgrounds and check if MCM10 expression differences cause differences in replication timing, genome stability or cell cycle defects. Finally, we need animal experiments and long-term clinical experiments for MCM10 small-molecule inhibitors, and then apply them to patients and use them in combination with ICI. Most importantly, we will solve these problems slowly, and our efforts have also achieved certain results. Therefore, future researches on Mcm10’s relationship to cancer development and progression may lead to discoveries with momentous prognostic and even therapeutic value.

## Data Availability

The datasets presented in this study can be found in online repositories. The names of the repository/repositories and accession number(s) can be found in the article/Supplementary Material.
